# Relatives’ Needs, Anxiety, and Expectations During the Intraoperative Waiting Period: A Cross-Sectional Survey in a Tertiary Referral Hospital in Morocco

**DOI:** 10.7759/cureus.104101

**Published:** 2026-02-23

**Authors:** Hamza Najout, Mohammed Moutaouakil, Ilyass Masad, Walid Atmani, Mustapha Bensghir

**Affiliations:** 1 Department of Anesthesiology and Intensive Care Unit (ICU), Mohammed V Military Teaching Hospital, Mohammed V University, Rabat, MAR

**Keywords:** anxiety, family-centered care, intraoperative waiting, patient satisfaction, peri-anesthesia communication

## Abstract

Background: Family-centered care is increasingly recognized as a core component of peri-anesthesia practice. However, relatives’ experiences during the intraoperative waiting period remain insufficiently documented, particularly in middle-income countries. Limited communication during surgery may be associated with heightened anxiety and dissatisfaction among family members, even in technically well-resourced hospitals.

Objective: The primary objective was to assess relatives’ anxiety levels and unmet needs during the intraoperative waiting period. Secondary objectives were to describe peri-anesthesia communication practices, sources of distress, and overall satisfaction with information provided at a high-volume tertiary referral hospital in Morocco.

Methods: A descriptive cross-sectional survey was conducted at Mohammed V Military Teaching Hospital (HMIM V-Rabat) over a four-month period. Adult relatives of patients undergoing surgery under anesthesia were invited to complete an anonymous, structured questionnaire after completion of surgery and initial postoperative communication. The survey explored sociodemographic characteristics, peri-anesthesia communication practices, emotional experience, and perceived unmet needs. Anxiety was assessed using a 5-point Likert scale, and analyses were strictly descriptive.

Results: A total of 500 relatives were included. High anxiety during the intraoperative waiting period was reported by 323 relatives (64.6%), while 92 (18.4%) reported moderate anxiety and 85 (17.0%) reported low anxiety. Most relatives, 389 (77.8%), received no information while surgery was ongoing, and only 165 (33.0%) were satisfied with the frequency of communication. The most frequently reported sources of anxiety were lack of information (70.4%), fear of surgical complications (62.4%), and prolonged waiting time (52.0%). Frequently identified unmet needs included more regular information updates (75.4%), access to a dedicated waiting area (72.2%), and the availability of a clearly identified contact person (65.2%).

Conclusion: Relatives’ experiences during the intraoperative waiting period represent an important yet under-addressed dimension of peri-anesthesia care in tertiary referral hospitals. High anxiety and dissatisfaction were frequently reported alongside communication and organizational constraints. Structured intraoperative information updates delivered through a designated contact person may represent a feasible and low-cost strategy for improving family-centered peri-anesthesia care.

## Introduction

Peri-anesthesia care has progressively evolved toward a family-centered model, acknowledging that the surgical journey extends beyond the patient to include relatives and close caregivers whose experiences are closely linked to the quality of care delivered. Family members frequently provide essential emotional support and contribute to postoperative recovery and continuity of care [1]. Family-centered care frameworks emphasize the inclusion of relatives as active participants in the perioperative process [2]. Despite this central role, the peri-anesthesia period remains a time of particular psychological vulnerability for relatives, especially during the intraoperative phase, when the patient is in the operating room and families must wait without direct contact or real-time clinical updates.

Evidence from peri-anesthesia and critical care literature indicates that insufficient or delayed communication while surgery is ongoing is associated with increased anxiety and emotional distress among relatives [3]. During the intraoperative waiting period, uncertainty regarding surgical progress and outcomes may contribute to heightened stress and a perceived loss of control [4]. Conversely, structured and compassionate communication delivered by peri-anesthesia teams has been associated with improved family satisfaction and enhanced trust in healthcare professionals [5]. Improved communication strategies have also been linked to higher levels of family satisfaction with care [6]. Although much of this evidence originates from intensive care settings, similar mechanisms of uncertainty, limited situational awareness, and reliance on clinical updates are likely relevant to the intraoperative waiting period, where families experience temporary separation from the patient without access to continuous information. Nevertheless, the intraoperative context differs from intensive care in duration, acuity, and relational dynamics, warranting context-specific investigation.

Despite increasing recognition of family-centered care principles, empirical data describing relatives’ needs and expectations during the intraoperative waiting period remain limited, particularly in low- and middle-income countries where peri-anesthesia communication practices have not been systematically evaluated [7]. In North African settings, including Morocco, published evidence specifically addressing relatives’ experiences while patients are in the operating room is scarce, limiting the transferability of findings derived predominantly from high-income healthcare systems [8].

These challenges may be further accentuated in military and teaching hospitals, which combine a broad spectrum of elective and emergency surgical activity with specific organizational characteristics, including strict security protocols, heterogeneous patient populations, and the dual academic and clinical responsibilities of healthcare professionals. In such environments, family satisfaction has been shown to be closely related to the clarity and accessibility of information provided by healthcare teams [6]. However, how these organizational dynamics shape relatives’ lived experiences during intraoperative waiting remains insufficiently documented.

At the Mohammed V Military Teaching Hospital (HMIM V) in Rabat, no prior evaluation has systematically examined the needs, expectations, or satisfaction of caregivers waiting while patients undergo surgery across different specialties. Given the volume and diversity of surgical activity at HMIM V, this institution provides a relevant and representative context for examining peri-anesthesia communication practices within healthcare environments characteristic of many North African referral centers.

The primary objective of the present study was to assess relatives’ anxiety levels and unmet needs during the intraoperative waiting period at HMIM V-Rabat. Secondary objectives were to describe peri-anesthesia communication practices, identify perceived sources of distress, and evaluate overall satisfaction with information provided during surgery. By generating locally grounded descriptive data, this study seeks to inform feasible, context-adapted strategies to enhance family-centered peri-anesthesia care in high-volume referral hospitals operating in resource-constrained environments.

## Materials and methods

Study design

This study was conducted as a descriptive, cross-sectional questionnaire survey designed to explore the needs, expectations, and experiences of family members during the intraoperative waiting period. For the purpose of this study, the intraoperative waiting period was defined as the time during which the patient was physically in the operating room, excluding the pre-anesthesia holding area, while relatives remained outside without direct contact or continuous clinical updates.

A cross-sectional descriptive design was selected to provide a structured snapshot of family perceptions within routine peri-anesthesia practice. The study was not designed to establish causal relationships or evaluate the effectiveness of specific communication interventions.

Setting

The study was conducted at HMIM V in Rabat, a tertiary military and academic referral center with a high volume of elective and emergency surgical activity. Surgical care includes general surgery, orthopedics, urology, visceral surgery, otorhinolaryngology, and other specialties. All procedures are performed in centralized operating rooms, followed by postoperative monitoring in a post-anesthesia care unit (PACU).

During the study period, there was no dedicated waiting area for relatives, and no formalized or standardized intraoperative family communication protocol was in place. Information delivery relied primarily on informal and non-systematized practices, typically depending on individual clinician initiative.

Participants

Participants were adult family members or designated close caregivers of patients undergoing surgery under anesthesia at HMIM V. Inclusion criteria were age ≥18 years, physical presence at the hospital throughout the intraoperative waiting period, and ability to understand and complete the questionnaire independently or with minimal clarification.

Family members of patients admitted directly to an intensive care unit prior to surgery and individuals who declined participation were excluded.

To ensure independence of observations and avoid clustering of responses related to a single surgical episode, only one relative per patient was included. When multiple relatives were present, the primary accompanying person or self-identified main contact was invited to participate. If this designation was unclear, the first eligible relative approached was included.

Data collection instrument

Data were collected using a structured, anonymous questionnaire developed specifically for this study after a focused review of literature addressing family needs, peri-anesthesia communication, and satisfaction with care.

The questionnaire comprised four domains: Sociodemographic characteristics of family members; Information and communication during the intraoperative waiting period; Emotional experience and sources of anxiety; Overall satisfaction and perceived unmet needs.

Anxiety was assessed using a single 5-point Likert scale item ranging from 1 (“no anxiety”) to 5 (“very high anxiety”). Predefined categories were established as follows: scores 1-2 = low anxiety, 3 = moderate anxiety, and 4-5 = high anxiety.

The instrument was reviewed by peri-anesthesia clinicians to assess content relevance and face validity. A pilot test was subsequently conducted with 10 family members representative of the study population to evaluate clarity and acceptability. Minor wording adjustments were made following pilot feedback.

Because the questionnaire explored heterogeneous domains rather than a single psychometric construct, formal internal consistency testing (e.g., Cronbach’s alpha) was not performed.

The questionnaire was administered in commonly used local clinical languages to ensure comprehension. A formal forward-back translation process was not undertaken, as the instrument was developed for exploratory local use rather than cross-cultural scale validation.

Data collection procedure

Data collection was performed after completion of the surgical procedure, once relatives had received initial postoperative information regarding the patient’s condition. This timing was chosen to avoid interfering with the waiting experience and to allow participants to respond in a more stable emotional state.

Questionnaires were completed in a self-report format. When necessary, limited assistance was provided for clarification of wording, without interpretation or influence on responses. Participation was voluntary, and no identifying information was recorded.

Waiting time was based on relatives’ self-reported perception rather than extraction from hospital timestamps, as the study aimed to evaluate subjective waiting experience.

Incomplete questionnaires were excluded from analysis. Partially completed forms were not retained.

Sample size and sampling strategy

Given the descriptive nature of the study, a pragmatic consecutive sampling approach was used. All eligible relatives present during the study period were invited to participate.

Data collection was conducted over a four-month period (August 1 to November 30), selected to capture routine elective and emergency surgical activity while avoiding major service disruptions. No formal a priori sample size calculation was performed, as the study was exploratory and not hypothesis-driven.

Data analysis

Data were entered into a spreadsheet and analyzed using IBM SPSS Statistics for Windows, Version 26 (Released 2018; IBM Corp., Armonk, New York, United States).

Analyses were strictly descriptive. Likert-type responses were treated as ordinal variables and summarized as frequencies and percentages. Categorical variables were reported as counts and proportions. Continuous variables, when applicable, were summarized using means and standard deviations or medians and interquartile ranges, depending on distribution.

No inferential statistical analyses or subgroup comparisons were performed, in keeping with the descriptive objectives of the study.

## Results

Participant flow

**Figure 1:**
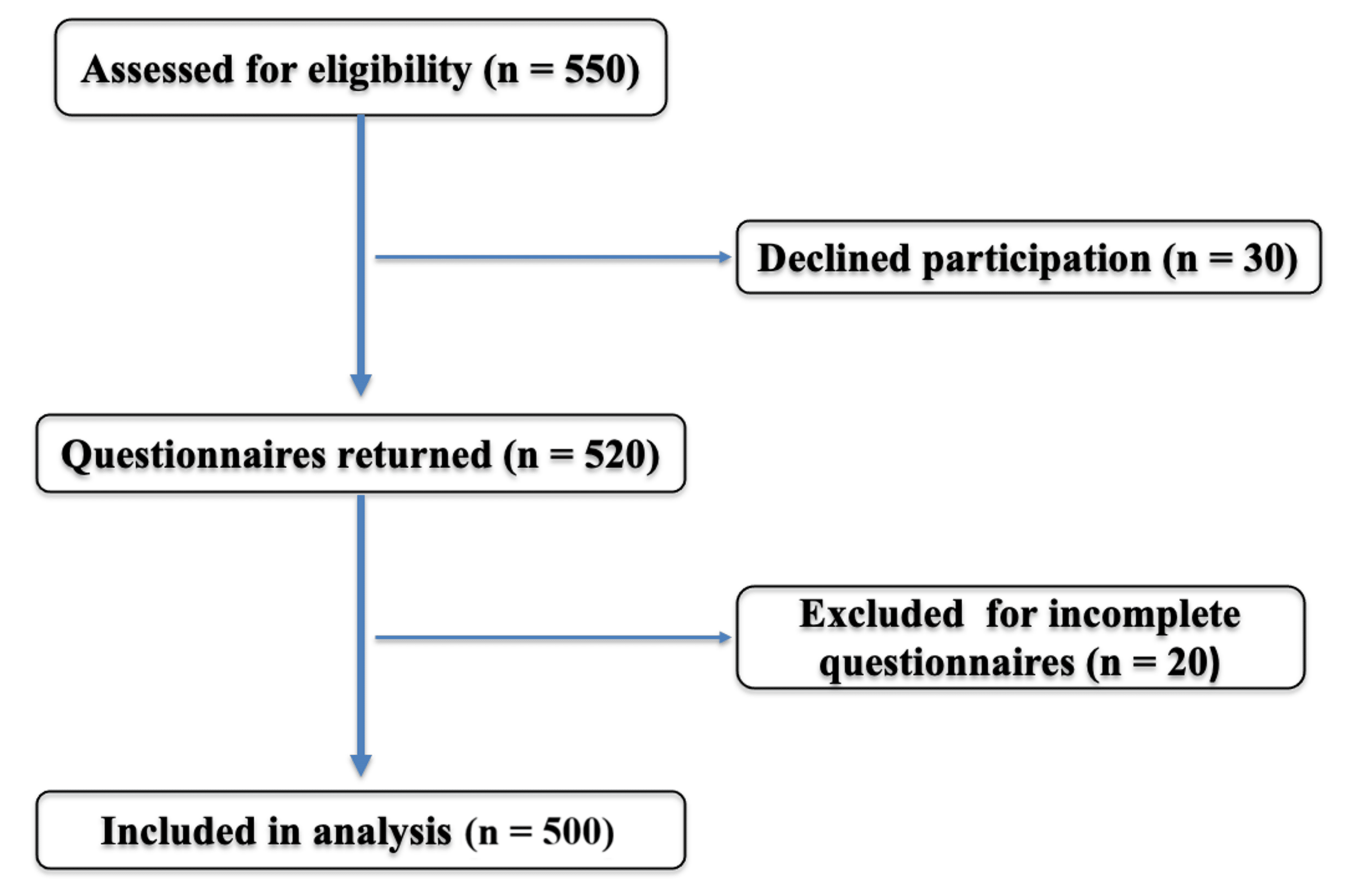
Flow diagram of participant inclusion Flow diagram illustrating assessment for eligibility, exclusions (declined participation and incomplete questionnaires), and final inclusion in the descriptive analysis.

During the study period, 550 family members were assessed for eligibility. Thirty individuals (5.5%) declined participation and 20 questionnaires (3.6%) were excluded due to incomplete responses. A total of 500 completed questionnaires were included in the final analysis, corresponding to a response rate of 90.9% among eligible participants (Figure 1).

Participant characteristics

Participants represented a broad age distribution. The largest age group was 40-49 years (153 participants, 30.6%), followed by 30-39 years (130, 26.0%). Respondents aged under 30 years accounted for 95 participants (19.0%), those aged 50-59 years for 87 (17.4%), and those aged ≥60 years for 35 (7.0%).

Male respondents constituted 280 participants (56.0%), while 220 (44.0%) were female.

Regarding relationship to the patient, spouses (175, 35.0%) and parents (145, 29.0%) were most frequently represented. Most participants (315, 63.0%) reported no previous experience of waiting during a surgical procedure.

Surgical activity encompassed multiple specialties. General surgery accounted for 180 cases (36.0%), orthopedic surgery for 103 (20.6%), urology for 87 (17.4%), and maxillofacial surgery for 85 (17.0%). Otolaryngology (30, 6.0%) and other specialties (15, 3.0%) represented smaller proportions (Table 1).

**Table 1:** Sociodemographic characteristics of participating relatives and distribution of surgical specialties This table presents age distribution, sex, and distribution of surgical specialties among the corresponding patients.

Variable	n	%
Age <30	95	19.0
Age 30–39	130	26.0
Age 40–49	153	30.6
Age 50–59	87	17.4
Age ≥60	35	7.0
Male	280	56.0
Female	220	44.0
General surgery	180	36.0
Orthopedic surgery	103	20.6
Urology	87	17.4
Maxillofacial surgery	85	17.0
Otolaryngology	30	6.0
Other surgery	15	3.0

Information and communication during the intraoperative waiting period

Preoperative information regarding expected surgical duration was reported by 206 participants (41.2%), whereas 294 (58.8%) indicated that no such information had been provided.

During surgery, 389 participants (77.8%) reported receiving no intraoperative information. Among those who received updates, information was delivered most frequently by surgeons (46, 9.2%), followed by nurses (28, 5.6%) and anesthesiologists (24, 4.8%). Twelve respondents (2.4%) reported receiving information from non-clinical staff (Table 2).

Self-reported waiting time exceeded two hours for 359 participants (71.8%), including 217 (43.4%) who waited between two and four hours and 142 (28.4%) who waited more than four hours. A total of 141 participants (28.2%) reported waiting less than two hours.

Satisfaction with the frequency of communication was reported by 150 participants (30.0%), while 230 (46.0%) expressed dissatisfaction. The remaining respondents reported neutral perceptions.

**Table 2:** Information and communication during the intraoperative waiting period This table describes preoperative information regarding expected surgical duration, sources of intraoperative updates, and self-reported waiting time among participating relatives.

Variable	n	%
No intraoperative information	389	77.8
Surgeon as an information source	46	9.2
Anesthesiologist as an information source	24	4.8
Nurse as an information source	28	5.6
Security staff as an information source	12	2.4
Waiting <2 h	141	28.2
Waiting 2–4 h	217	43.4
Waiting >4 h	142	28.4

Emotional experience, satisfaction, and unmet needs

Anxiety during the intraoperative waiting period was assessed using a 5-point Likert scale. High anxiety (scores 4-5) was reported by 323 participants (64.6%), moderate anxiety (score 3) by 92 (18.4%), and low anxiety (scores 1-2) by 85 (17.0%).

The most frequently reported sources of anxiety were lack of information (352, 70.4%), fear of surgical complications (312, 62.4%), and prolonged waiting time (260, 52.0%).

Perceived support from healthcare staff was rated as adequate or very adequate by 165 participants (33.0%), whereas 205 (41.0%) considered support inadequate or very inadequate. Overall satisfaction with communication during the waiting period was reported by 150 respondents (30.0%), while 210 (42.0%) expressed dissatisfaction.

Commonly identified unmet needs included more frequent information updates (377, 75.4%), access to a dedicated waiting area (361, 72.2%), availability of a clearly identified contact person (326, 65.2%), and improved basic comfort measures such as seating and access to water (290, 58.0%) (Table 3).

**Table 3:** Emotional experience, satisfaction, and unmet needs during the intraoperative waiting period This table summarizes anxiety levels assessed using a 5-point Likert scale, reported sources of distress, perceived adequacy of staff support, and the most frequently identified unmet needs among participating relatives.

Variable	n	%
High anxiety (Likert 4–5)	323	64.6
Lack of information (anxiety source)	352	70.4
Fear of complications	312	62.4
Need more frequent information	377	75.4
Need waiting area	361	72.2
Need designated contact person	326	65.2

## Discussion

This study provides a detailed insight into the experiences of relatives during the intraoperative waiting period in a high-volume tertiary referral hospital. Although surgical care at HMIM V-Rabat is delivered in a technically advanced environment, our findings indicate that family members frequently report substantial anxiety and low satisfaction with communication. These observations suggest that organizational and communicational dimensions of peri-anesthesia care represent an essential complement to technical performance in shaping the overall family experience.

The high prevalence of anxiety observed in our cohort is consistent with previous findings from peri-anesthesia and critical care settings, where uncertainty and lack of timely information are repeatedly identified as major sources of psychological distress [3]. Similar emotional responses have been described among relatives of critically ill patients, suggesting that the intraoperative waiting period may generate levels of anxiety approaching those observed in intensive care contexts, even when patients undergo elective procedures [4]. Although much of the available literature originates from intensive care environments, the underlying mechanisms, uncertainty, dependence on clinical updates, and temporary separation from the patient are conceptually comparable to those experienced during intraoperative waiting.

In our dataset, higher anxiety levels appeared associated with limited access to real-time information while surgery was ongoing, reinforcing the perception of reduced situational awareness frequently described in qualitative research [9]. However, given the descriptive design and absence of inferential analyses, these associations should not be interpreted as evidence of causality. Rather, they highlight patterns warranting further analytical investigation. Qualitative studies have similarly emphasized that communication quality strongly influences relatives’ emotional responses during acute care episodes [10,11].

Dissatisfaction in our cohort appeared predominantly associated with reported communication and organizational characteristics. More than three-quarters of relatives indicated that they received no intraoperative information, and fewer than one-third were satisfied with the frequency of updates. In contrast, healthcare systems that have implemented structured perioperative communication strategies, such as scheduled updates, perioperative liaison personnel, or dedicated waiting areas, have reported improvements in family satisfaction and reductions in perceived distress [12]. Prior studies indicate that family satisfaction correlates with clarity, accessibility, and consistency of communication rather than procedural complexity alone [13].

In this context, our findings may reflect the absence of formalized communication pathways within routine peri-anesthesia workflows, rather than limitations in technical capability or clinical expertise. Similar organizational gaps have been described in high-volume referral hospitals internationally, where intense clinical activity often results in communication being managed on an ad hoc basis [14,15]. Importantly, contemporary anesthesiology literature increasingly recognizes structured perioperative communication as an integral component of patient safety, quality of care, and team performance.

The institutional setting of HMIM V-Rabat adds further interpretative nuance. As a national military and teaching referral center, the hospital operates under specific structural characteristics, including security constraints, hierarchical organization, and dual clinical-academic responsibilities. Such complexity may influence communication dynamics independently of resource availability. Distinguishing structural organization from clinical competence is therefore essential when interpreting dissatisfaction patterns.

The military nature of the institution may also subtly shape family-staff interactions. Hierarchical environments can discourage relatives from proactively requesting updates or expressing concerns, particularly where institutional formality is perceived as normative. Because this dimension was not directly measured, it should be regarded as a contextual hypothesis rather than an empirically demonstrated explanatory factor. Comparable influences of institutional culture on communication behaviors have been described in acute care decision-making frameworks [16]. These contextual elements may contribute to persistent uncertainty during intraoperative waiting.

Beyond anxiety, relatives consistently identified unmet needs related to information accessibility, predictability of updates, and environmental comfort. Requests for more frequent communication, a clearly designated contact person, and access to a dedicated waiting area align closely with established family-centered care principles emphasizing transparency and relational continuity [10]. Environmental conditions and communication clarity have also been associated with perceived care quality across diverse healthcare systems [17]. Importantly, many proposed improvements are organizational rather than technological in nature, suggesting that incremental adjustments may yield meaningful gains in family experience without substantial financial investment [18].

The originality of this investigation lies in both its scale and contextual focus. To our knowledge, this represents the first large-scale study in Morocco specifically examining relatives’ experiences during the intraoperative waiting period, with 500 participants across multiple surgical specialties. Given the limited regional data, these findings contribute descriptive evidence relevant to tertiary referral centers operating within comparable North African healthcare environments.

Several limitations merit careful consideration. The questionnaire was developed specifically for this exploratory study and was not subjected to formal psychometric validation. Although items were informed by existing literature and refined through expert review and pilot testing to ensure contextual relevance, comparability with standardized anxiety instruments remains limited. The single-center, cross-sectional design restricts external generalizability and precludes causal inference. Social desirability bias cannot be excluded.

Furthermore, questionnaires were completed after relatives received initial postoperative information, introducing the possibility of outcome-dependent perception bias. Positive or negative clinical news may have influenced retrospective appraisal of the waiting experience. In addition, the absence of subgroup analyses (e.g., by surgical specialty, elective versus emergency procedures, or relationship to the patient) limits exploration of potential modifiers of anxiety and satisfaction.

From a practical perspective, the study identifies feasible opportunities for improvement. Potential interventions include the introduction of a concise informational leaflet outlining the perioperative pathway, designation of a staff member responsible for scheduled intraoperative updates, and brief communication-focused training initiatives for junior physicians. Such strategies are low-cost and consistent with evidence suggesting that structured communication enhances family satisfaction in perioperative contexts [15,19].

Future research would benefit from mixed-methods designs combining quantitative assessment with qualitative exploration of relatives’ lived experiences, as well as prospective interventional studies evaluating the impact of structured communication pathways in comparable institutional settings.

## Conclusions

Relatives’ experiences during the intraoperative waiting period represent an important yet frequently overlooked dimension of peri-anesthesia care in high-volume tertiary referral hospitals. In this descriptive cross-sectional survey, a substantial proportion of family members reported high levels of anxiety and dissatisfaction with intraoperative communication. These findings suggest that communication practices and organizational structures may play a central role in shaping the family experience during surgery.

Although causal relationships cannot be inferred, the consistency of reported unmet needs highlights potential opportunities for improvement. Structured intraoperative information updates, identification of a designated contact person, and clearer communication pathways may represent feasible, low-cost strategies to enhance family-centered peri-anesthesia care. Future research combining qualitative exploration and prospective interventional evaluation may help determine the effectiveness of structured communication models in similar institutional settings.

## Appendices

Questionnaire

https://docs.google.com/document/d/18txnUqZXIuY9iU4DOBfudYB6y-D4BpMZ/edit
